# Underfunding Basic Psychological Science Because of the Primacy of the Here and Now: A Scientific Conundrum

**DOI:** 10.1177/17456916221105213

**Published:** 2022-09-06

**Authors:** Jorge Almeida

**Affiliations:** 1 Proaction Lab, Faculty of Psychology and Educational Sciences, University of Coimbra; 2 CINEICC, Faculty of Psychology and Educational Sciences, University of Coimbra

**Keywords:** basic science, funding

## Abstract

The psychological sciences are suffering from the primacy of the “here and now” and from a radical utilitarian view of science—this is certainly true in less affluent countries around the world. Portugal is a particular case study, as both psychology departments and funding agencies are largely biased toward applied psychology—but this is a more global trend. The field needs to find a balance between applied and basic psychology to better respond to the challenges of tomorrow.

One of the things that the current pandemic has taught us is that, in the long run, underfunding basic science can have dire consequences on the limits and strength with which societies can respond to pressing global challenges. For instance, it is certainly not hard to imagine that we would be much worse off had we not funded years and years of basic research in the biological sciences that then allowed for the record-breaking generation of vaccines for COVID-19.

But basic biological sciences are not alone in shaping and majorly impacting society. The field of basic psychology (including experimental, social and cognitive psychology, cognitive and affective neuroscience, comparative psychology, etc.) has also been fertile in impacting society throughout the years. A major example comes from the work of Nobel laureate Daniel Kahneman and his longtime collaborator Amos Tversky on human (ir)rationality. Their understanding of humans as users of heuristics in decision-making and their proclivity to fall prey to fallacies and systematic errors when making decisions (e.g., Tversky & Kahneman, 1974) has been a game changer for most social sciences and for society at large. It is perhaps hard now to envision modern life without these fundamental contributions. Indeed, Kahneman and Tversky’s basic work has influenced economic theories, philosophy, and global perspectives on health and may figure centrally in our efforts to understand change and individual and group decisions about topics as important as climate change. Other excellent examples of how basic psychological research contributed greatly to our society come, for instance, from the work on language acquisition and face recognition. The work of many psycholinguists on the basic processes of language acquisition has been paramount for our capacity to intervene in the early education of linguistic skills (e.g., Snow, 1992); finally, current major commercial artificially intelligent face-recognition algorithms have been highly influenced by the ideas and models of face recognition coming from cognitive psychology (e.g., Biederman & Kalocsais, 1997).

However, and perhaps surprisingly given the above, we live under the primacy of the “here and now” in science (i.e., under a radical utilitarian view of science), with a major bias toward looking for, and funding, immediate solutions for applied problems, mostly at the expense of funding basic science and thus at the expense of putting forth the scaffolding for safe and efficient applied responses to today’s (and tomorrow’s) societal challenges. One key example of this zeitgeist was the discussion around a major cutting of the European Research Council’s (ERC) budget and thus its support of groundbreaking basic research (“A pandemic is no time to cut the European Research Council’s funding,” 2020). In fact, the threat to science and society that underfunding basic science poses has been clearly exposed in major position statements such as *Science Report: Towards 2030* (United Nations Educational, Scientific and Cultural Organization [UNESCO], 2015), the International Council for Science’s statement (International Council for Science [ICSU], 2004), or the proclamation by the United Nations of 2022 as the International Year of Basic Sciences for Sustainable Development (https://www.iybssd2022.org/en/home). These reports show that there is clear underfunding of basic science, and that that will inevitably lead to crippling the applied sciences and the capacity to respond to pressing societal challenges.

Moreover, defunding basic science, and the push for the fast and immediate application of science, in lieu of applied solutions based on well-funded basic science will also have major consequences for science itself. For instance, in the field of psychology many authors have raised awareness to the increased reliance on concepts that are not yet mature enough and/or to the resistance by students and practitioners of relying on the scientific method and scientific evidence (e.g., Baker et al., 2008; Ferguson, 2015; Lilienfeld, 2012). According to these authors, many practitioners and students in the field of psychology fail to see the relevance of basic psychological science to their own practice and future. These authors (e.g., Baker et al., 2008; Ferguson, 2015; Lilienfeld, 2012) suggest, among other things, the need to rebrand psychology, in the eyes of society, psychologists and students of psychology, also as a basic science, psychology should focus on understanding human behavior through scientific methodologies. They also advocate the need to measure the progress in psychology (at least) not exclusively on how the psychological sciences immediately resolve mental-health issues and other pressing societal challenges, but also on how our basic understanding of the human mind advances.

These discussions around cutting the budgets of programs that support basic research and the focus on immediacy and a radical utilitarian view of science may come to be because policymakers, and perhaps society at large, have a harder time realizing the central (but less immediate) role that basic research plays on overall science and on facilitating solutions for societal problems. This is not a novel problem, however. In a compelling and poignant essay more than 80 years ago, Abraham Flexner (1939) exposed a dangerous trend for utilitarianism in science and advocated for purportedly “useless” (basic) science by unequivocally showing that many of the major scientific applications of the time (as in our time) were based on years and years of (nurtured and funded) work in basic science.

As Flexner suggested, it is thus the role of universities, the science community, and national and international funding agencies, as strongholds of science, to strive for a balance between applied and basic research. This is because they (should) understand, unequivocally, that this balance is key for the sustained and efficient development of science (basic or applied). Again, one key example of how these strongholds should defend science came from ERC’s former president Jean-Pierre Bourguignon with his role as a fierce advocate for funding basic science and for adequate budgeting for the ERC.

Unfortunately, the pulling of the rug under basic science’s feet still percolates to (at least) some national science foundations and academic institutions—this is certainly true (with very few exceptions) in many of the less affluent countries (e.g., some Southern and Eastern European countries, South American countries). One major example of this is Portugal, and a research field in which this is especially true is the psychological sciences. The psychological sciences are a major case study because in this field of research there is a somewhat clear division between applied and basic research and because there are particularly hard challenges ahead in mental health because of the pandemic and climate change that will require both basic and applied psychological-science research.

Nevertheless, in the last 3 to 4 decades, all major (public and private) psychology departments in Portugal have clearly been dominated by, and have mainly fostered, applied subareas of psychology. Specifically, around 60% of the tenured faculty at Portuguese psychology departments have research interests revolving exclusively around clinical and mental health topics, and probably about 80 to 90% of the faculty focuses on applied psychology areas (e.g., clinical psychology, work, organizational and personnel psychology, educational psychology). The remaining faculty includes statisticians, biologists, and (very few) researchers dedicated to basic psychology and cognition. Despite the now distant cognitive revolution of the 1950s and 1960s, the consecutive decades of the brain and the mind of the 1990s and early 2000s, and even the fact that the curricula of most BA psychology programs in Portugal include some (perhaps few) typical cognitive and neuroscientific courses, there are still very few faculty whose research interests fall within cognitive, social, and developmental basic psychology, cognitive neuroscience is still a foreign body to these departments, and comparative psychology is almost extinct. That is, psychology departments in Portugal ignore basic research, having fewer than five (of about 60) faculty on average dedicated to basic psychology.

This is in stark contrast with how top universities and psychology departments in the world manage the balance between basic and applied science. Specifically, the highest ranked psychology departments in the world (according to most if not all of the international rankings; e.g., Harvard University; Yale University; Stanford University; Massachusetts Institute of Technology; University of California, Los Angeles; University College London; Oxford University; Princeton University; University of California, Berkeley, among others) tend to present a prevalence of basic psychological scientists (or at the very least a balance between the number of basic and applied psychology scientists) in their faculty, along with a clear focus on neuroscientific approaches to psychology and cognition. Moreover, applied subareas within these departments are very much influenced by the strong basic research core. This focus on basic psychology and on neuroscientific approaches to the mind does not happen by mere chance or accident—these are carefully considered efforts because these departments also have to fight against the policymakers’ need for immediate solutions. Furthermore, this balance and scientific core figures critically in the leadership of these universities and departments when it comes to the psychological sciences.

The challenges for the psychological sciences in Portugal have recently been amplified because the Portuguese Foundation for Science and Technology (the major national funding agency for science) has also failed to implement a balance between applied and basic research in the psychological sciences. The pinnacle of this failure was the selection of the members of one of the latest evaluation panels for the annual call for project grants in all scientific domains. The psychology panel was composed of 15 international researchers, eight of whom were researchers with research interests clearly within the clinical and mental health domain. Of the remaining seven, four were researchers with applied (nonclinical) research interests, and only three were researchers dedicated to basic psychology. This unbearable imbalance had clear consequences on the kinds of projects that were funded: Of the five projects selected for funding, four focused on applied psychology topics. These five projects were selected out of 101 projects, in which more than a third focused on basic psychology topics. The similarity between the ratio of applied-to-basic scientists in the evaluation panel and applied-to-basic projects selected is uncanny. Moreover, and perhaps more distressing, is that many of the basic psychology projects received commentaries in their evaluation reports that pointed to the lack of applied science topics and applied psychologists as team members in the proposals.

This is the problem we deal with in the psychological sciences in Portugal, and many, if not most, of the less affluent countries in the world. Thus, this is a global problem and threat: When all of the strongholds of science (science foundations and universities) ostensibly ignore the importance of basic research in psychology (and other areas), then we are left unprotected against (scientific) populism; when the very same institutions that should defend basic science are those that perpetuate the imbalance and foster the primacy of the here and now and a radical utilitarianism in science at the expense of a balanced focus on both applied and basic psychology, then we will, in the near future, suffer from a lack of basic scientific knowledge to support fast and effective applied solutions to novel and unexpected emergent problems.

This is the conundrum—by defunding basic science to uniquely or even predominantly support the here and now and applied science, we will end up with problems in resolving the very same (applied) issues we were trying to prioritize to begin with.

Perhaps not all is lost, however, as can be seen in how the positions of the ERC, UNESCO, the ICSU, or the United Nations, unlike the national science foundation in Portugal (along with other national science foundations), fought for basic science despite the zeitgeist, or in how major psychology departments promote basic psychology and neuroscientific approaches and thus facilitate strong applied psychology based on the scaffolding of basic psychological science.

It is clear that researchers in psychology (not only in Portugal and in less affluent countries but also globally) need to think carefully about where to go as a field and whether we really want to ignore the importance of basic research. Science stakeholders overall need to understand that science-based approaches to societal challenges (whether it is mental health after the pandemic or climate change in the next decades) need to be well funded, and that the solutions for the problems of the here and now necessitate, or at least strongly benefit from, investing in basic research. This can only happen if those in charge of major funding agencies and of major universities and (in this case) psychology departments realize that the future is now. Funding and fostering basic and applied psychology alike will impact not only our ability to deal with the major mental-health issues resulting from COVID-19 but also all major challenges that we will face in the near and not-so-near future.

## Transparency

*Action Editor:* Tina M. Lowrey

*Editor:* Klaus Fiedler
